# Human stool contains a previously unrecognized diversity of novel astroviruses

**DOI:** 10.1186/1743-422X-6-161

**Published:** 2009-10-08

**Authors:** Stacy R Finkbeiner, Lori R Holtz, Yanfang Jiang, Priya Rajendran, Carl J Franz, Guoyan Zhao, Gagandeep Kang, David Wang

**Affiliations:** 1 Departments of Molecular Microbiology and Pathology & Immunology, Washington University School of Medicine, St Louis, MO USA; 2 Department of Pediatrics, Washington University School of Medicine, St Louis, MO USA; 3 Department of Gastrointestinal Sciences, Christian Medical College, Vellore, India

## Abstract

Human astroviruses are a leading cause of gastrointestinal disease. Since their discovery in 1975, 8 closely related serotypes have been described in humans, and more recently, two new astrovirus species, astrovirus MLB1 and astrovirus VA1, were identified in diarrhea patients. In this study, we used consensus astrovirus primers targeting the RNA polymerase to define the diversity of astroviruses present in pediatric patients with diarrhea on two continents. From 416 stool specimens comprising two different cohorts from Vellore, India, 35 samples were positive. These positive samples were analyzed further by either sequencing of the ~400 bp amplicon generated by the consensus PCR or by performing additional RT-PCR specific for individual astroviruses. 19 samples contained the classic human astrovirus serotypes 1-8 while 7 samples were positive for the recently described astrovirus MLB1. Strikingly, from samples that were positive in the consensus PCR screen but negative in the specific PCR assays, five samples contained sequences that were highly divergent from all previously described astroviruses. Sequence analysis suggested that three novel astroviruses, tentatively named astroviruses VA2, MLB2 and VA3, were present in these five patient specimens (AstV-VA2 in 2 patients, AstV-MLB2 in 2 patients and AstV-VA3 in one patient). Using the same RT-PCR screening strategy, 13 samples out of 466 tested stool specimens collected in St. Louis, USA were positive. Nine samples were positive for the classic human astroviruses. One sample was positive for AstV-VA2, and 3 samples were positive for AstV-MLB2 demonstrating that these two viruses are globally widespread. Collectively, these findings underscore the tremendous diversity of astroviruses present in fecal specimens from diarrhea patients. Given that a significant fraction of diarrhea etiologies is currently unknown, it is plausible that these or other yet unrecognized astroviruses may be responsible for at least part of the undiagnosed cases.

## Findings

It is estimated that diarrhea results in 2 million deaths and 1.4 billion non-fatal cases each year, primarily in young children [1,2]. The viruses that cause the greatest proportion of diarrhea are rotaviruses, caliciviruses, adenoviruses and astroviruses [3-7]. However, while there are many known agents of diarrhea, an etiologic agent cannot be found in ~40% of diarrhea cases according to most epidemiological studies [8-10].

Astroviruses are known to infect a variety of human and animal hosts. Astroviruses were first discovered in humans in 1975 [11]. Until 2008, human infections were thought to be limited to 8 closely related serotypes (hereafter referred to as "classic" human astroviruses). We recently identified two highly divergent members of the astrovirus family, astrovirus MLB1 (AstV-MLB1) [12-14] and astrovirus VA1 (AstV-VA1) [15], in patients with sporadic diarrhea and patients from a gastroenteritis outbreak, respectively. In parallel, a spate of new astrovirus species have also been detected recently in dogs, cheetahs, sea lions, dolphins and bats [16-20]. Strikingly, more than 100 genetically distinct astroviruses have been detected in different bat species[17,20].

In humans, astroviruses predominantly affect children under the age of 2, the elderly, and immunocompromised individuals [21]. Up to ~10% of sporadic cases of non-bacterial diarrhea in children are attributed to the classic human astroviruses [4,5,22-24]. No definitive disease association has been established yet for the recently discovered AstV-MLB1 or AstV-VA1.

Following the discovery and complete genome sequencing of AstV-MLB1, we designed consensus PCR primers in order to detect both classic astroviruses and AstV-MLB1 in a single assay [14]. In the current study, this astrovirus consensus PCR assay was used to define the diversity of astroviruses present in pediatric patients with diarrhea in Vellore, India and St. Louis, USA. We report not only the first description of AstV-MLB1 in India, but also the detection of three additional distinct and highly divergent novel astroviruses.

Two sets of approximately 200 samples each (n = 416) collected in 2005 and 2006 in Vellore, India were tested in this study. The first set of community-based samples was from a birth cohort. The cohort of 452 children was recruited between 2002 and 2003, followed for 3 years with twice-weekly home visits and collection of stool during every diarrheal episode (n = 1955). The second set of samples was from single point collection of diarrheal stool from hospital based surveillance for children under the age of 5 years hospitalized for acute gastroenteritis in 3 hospitals in India from 2005 to 2007. For both community and hospital based studies, the severity of the diarrheal episode was recorded using the 20 point Vesikari scale developed for rotaviral gastroenteritis, which includes number and duration of diarrhea and vomiting episodes, presence of fever and dehydration and classifies gastroenteritis as mild, moderate, severe and very severe [25]. The samples tested were selected at random from these cohorts, with equal numbers taken from each month over a 12-month period. In St. Louis, 466 stool specimens that were sent for bacterial culture to the clinical microbiology laboratory at the St. Louis Children's hospital between June 2008 and March 2009 were analyzed.

For each sample from Vellore, 200 μL of a ~20% fecal suspension were extracted using the Boom method [26] and the extracted RNA was eluted into 40 μL of water. The St. Louis stool samples were diluted at a 1:6 ratio (wt/vol) in phosphate-buffered saline and then RNA was extracted from 330 μL of each sample using a COBAS Ampliprep Version 3.1.0 (ROCHE, Indianapolis, IN). The RNA was eluted in a final volume of 200 μL. A two-phase screening strategy to detect and identify astroviruses was used. In the first phase, the Qiagen OneStep RT-PCR kit (Qiagen, Valencia, CA) was used to screen 3 μL of extracted material from each sample using the consensus primers SF0073 (5'-GATTGGACTCGATTTGATGG-3') and SF0076 (5'-CTGGCTTAACCCACATTCC-3') that target highly conserved regions in the ORF 1b (RNA polymerase) of astroviruses. Samples that were positive in the first round of screening were then subjected to additional RT-PCR screenings with primers specific for classic human astrovirus [Mon269 (5'-CAACTCAGGAAACAGGGTGT-3') and Mon270 (5'-TCAGATGCATTGTCATTGGT-3')] [27], AstV-MLB1 [SF0053 (5'-CTGTAGCTCGTGTTAGTCTTAACA-3') and SF0061 (5'-GTTCATTGGCACCATCAGAAC-3')], and AstV-VA1 [SF0178 (5'-GCTGTCACCGTCTCTGCCACCAT-3') and SF0179 (5'-TCTACATACAAGGATGCAGCATG-3')], using the QIAGEN One-Step RT-PCR kit under the following cycling conditions: 30 min RT step, 94°C hold for 10 min, followed by 40 cycles of 94°C for 30 s, 56°C for 30 s, and 72°C for 50 s.

**Figure 1:**
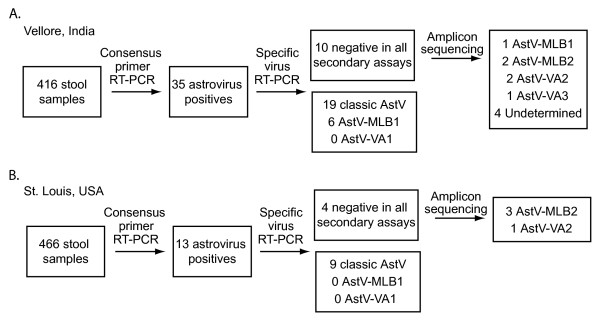
**Schematic of RT-PCR screening strategy and results**. Summary of astrovirus RT-PCR screening in A) Vellore, India and B) St. Louis, USA.

**Figure 2:**
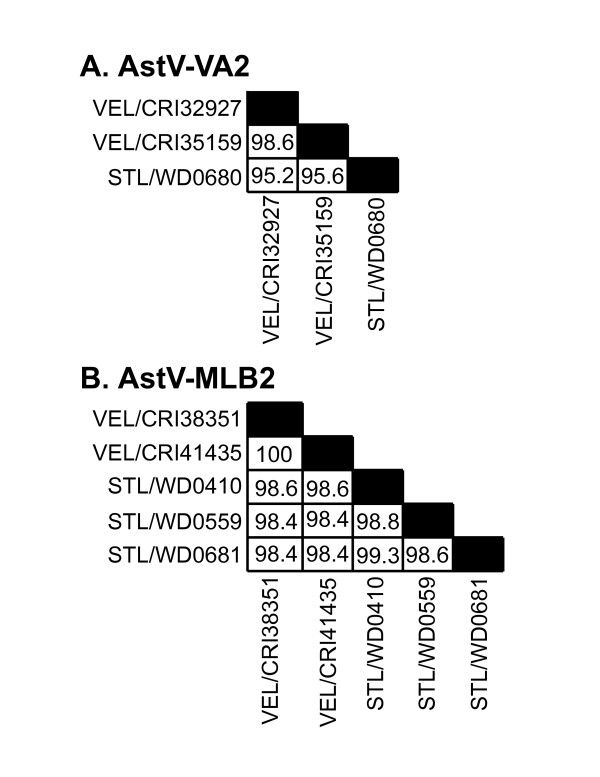
**Comparison of sequence identities between isolates**. Sequence identities between isolates of A) AstV-VA2 and B) AstV-MLB2 were calculated for the region of ORF1b amplified by the consensus primers using the DNAstar program.

Of the total 416 samples screened in Vellore, India, 35 tested positive with the astrovirus consensus primers (Figure 1). In the second phase of RT-PCR screening, 19/35 tested positive for classic human astroviruses, while an additional 6/35 tested positive for AstV-MLB1 (Figure 1). The remaining 10 samples were negative in specific classic human astrovirus, AstV-MLB1, and AstV-VA1 assays. Four of the samples could not be cloned despite repeated attempts and were not further characterized. We were able to clone and sequence the ~400 bp amplicons generated by the consensus primers in the other 6 samples. One of these samples was 97% identical at the amino acid level to the reference AstV-MLB1 sequence in Genbank. The fact that this virus was not detected by the AstV-MLB1 specific primers in the second phase of screening may be due to sensitivity issues or possibly the result of additional sequence variation in this isolate. Strikingly, three distinct sequences highly divergent from previously described astroviruses were detected in the other 5 samples. In two of the samples, the amplicons shared 68.5 and 69.2% amino acid identity to AstV-VA1. This virus has provisionally been named astrovirus VA2 (AstV-VA2). The two AstV-VA2 sequences were 98.6% identical to each other at the nucleotide level (Figure 2). The second virus, tentatively named astrovirus VA3 (AstV-VA3), was detected in one sample and shared 73.3% identity to AstV-VA1 and 71.9-72.6% identity to AstV-VA2. Finally, the two remaining samples had sequences that shared 80.8% amino acid identity to AstV-MLB1 and shared 100% nucleotide identity to each other. This virus has tentatively been named astrovirus MLB2 (AstV-MLB2).

All of the samples which were positive for AstV-MLB1, AstV-MLB2, AstV-VA2, and AstV-VA3 in Vellore were obtained from the community based studies. In the samples positive for these viruses, the diarrheal episodes were mainly mild or moderate, with only two episodes classified as severe based on Vesikari score [25]. All children recovered from their illness within a week at most, with only 4 episodes lasting longer than 3 days.

Of the 466 samples screened from St. Louis, MO, 13 tested positive with the astrovirus consensus primers (Figure 1). In the second phase of RT-PCR screening, 9/13 tested positive for classic human astroviruses, while none of them tested positive for AstV-MLB1 or AstV-VA1 (Figure 1). The amplicons generated by the consensus primers from the remaining 4 samples that were negative in the second phase RT-PCR assays were sequenced. AstV-VA2 was detected in 1 of the samples and AstV-MLB2 was detected in the remaining 3 samples demonstrating that both of these viruses are present in both India and the United States. Sequence comparisons of all isolates of AstV-VA2 and AstV-MLB2 from both Vellore and Saint Louis are shown in Figure 2.

To further characterize these viruses, RNA extracted from one stool sample positive for AstV-VA2 and one sample positive for AstV-MLB2 was sequenced using a combination of RT-PCR and pyrosequencing on a Roche Genome Sequencer as described [15]. Contigs of 5977 bp for AstV-VA2 and 3980 bp for AstV-MLB2 were assembled and then confirmed by generating a series of overlapping amplicons by RT-PCR. All sequences have been deposited in Genbank [MLB2: GQ502188-GQ502192; VA2: GQ502193-GQ502195; VA3: GQ502196]

**Figure 3:**
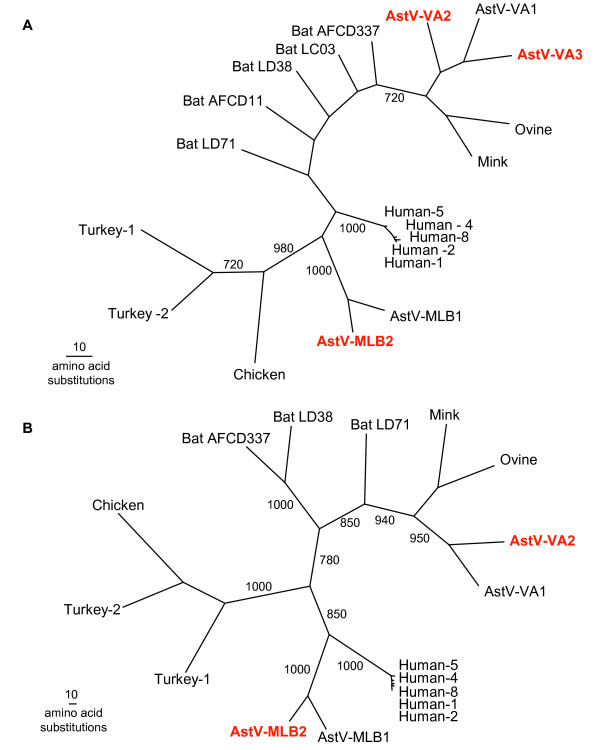
**Phylogenetic analysis of novel astroviruses**. A) Phylogenetic tree of the ORF1b amplicon generated by the consensus astrovirus primers. Multiple sequence alignments were then generated with these sequences and the corresponding regions of known astroviruses using ClustalX. PAUP was used to generate phylogenetic trees and bootstrap values (>700) from 1,000 replicates are shown. B) Phylogenetic analysis of the complete predicted ORF1b.

Phylogenetic analysis was carried out initially with the consensus amplicon sequences from each of the new viruses along with astrovirus ORF1b sequences available in GenBank using ClustalX1.83. PAUP was used to generate maximum parsimony trees with 1,000 bootstrap replicates [28]. The phylogenetic analysis clearly indicated that even based on this highly conserved region of the astrovirus genome, these three new viruses are highly divergent from any of the previously known astroviruses (Figure 3A). A phylogenetic tree using the complete ORF1b sequence from both AstV-VA2 and AstV-MLB2, along with available complete ORF1b sequences in Genbank gave similar results (Figure 3B). The topology of these trees suggests that there are multiple clades of astroviruses present in humans: the classic human astrovirus clade, a second clade of MLB1-like viruses and a third clade of VA1-like viruses.

In this report, consensus astrovirus primers demonstrated that AstV-MLB1 is present in India. This observation expands the known geographic range of AstV-MLB1 beyond Australia and North America to now include Asia. Unexpectedly, consensus astrovirus RT-PCR screening also demonstrated sequences from three highly divergent novel astroviruses were present in the Indian samples, two of which were also detected in St. Louis. This study, coupled with the recent discoveries of AstV-MLB1 and AstV-VA1, demonstrate that there is a much greater diversity of astroviruses that can be found in human stool than the 8 classic human astroviruses. Furthermore, a very recent report described 3 distinct astroviruses most closely related to ovine and mink astroviruses, which may be similar to AstV-VA1, AstV-VA2 and AstV-VA3 [29]. As sequences from that report are not yet available in Genbank, we have not been able to make definitive comparisons. The detection of these viruses in human stool may be the result of either non-infectious dietary ingestion or bona fide human infection. The role these three new viruses play in diarrhea or other human diseases is currently unclear; however, given the fact that most known members of the *Astroviridae *family, regardless of host, can cause diarrheal disease, it is tempting to speculate that one or more of these viruses may be responsible for some fraction of the estimated 40% of diarrhea cases of unknown etiology. Further experimentation is needed to define the role of these viruses in diarrhea or other human diseases.

## Competing interests

The authors declare that they have no competing interests.

## Authors' contributions

SF helped design and carried out experiments and analysis and wrote the manuscript. LH carried out experiments and analysis. YJ carried out experiments and analysis. PR helped carry out experiments and provided reagents. CF performed experiments. GZ analyzed data. GK provided samples. GK and DW conceived and designed the experiments and helped write the manuscript. All authors have read and approved the manuscript.
